# High chromosome instability identified by low-pass whole-genome sequencing assay is associated with *TP53* copy loss and worse prognosis in *BRCA1* germline mutation breast cancer

**DOI:** 10.1007/s12282-021-01286-1

**Published:** 2021-08-17

**Authors:** Liang Zhu, Jia-Ni Pan, Ziliang Qian, Wei-Wu Ye, Xiao-Jia Wang, Wen-Ming Cao

**Affiliations:** 1 Department of Pathology, Cancer Hospital of the University of Chinese Academy of Sciences (Zhejiang Cancer Hospital), Hangzhou, 310022 China; 2 Institute of Cancer and Basic Medicine (ICBM), Chinese Academy of Sciences, Hangzhou, 310022 China; 3 Department of Breast Medical Oncology, Cancer Hospital of the University of Chinese Academy of Sciences (Zhejiang Cancer Hospital), Hangzhou, 310022 China; 4 Zhejiang Chinese Medical University, Hangzhou, 310053 China; 5 Prophet Genomics Inc, San Jose, CA 95131 USA; 6 Suzhou Hongyuan Biotech Inc, Biobay, Suzhou, 215123 China

**Keywords:** CIN, *TP53*, *BRCA1*, LPWGS, Breast cancer

## Abstract

**Background:**

Though *BRCA1* mutation is the most susceptible factor of breast cancer, its prognostic value is disputable. Here in this study, we use a novel method which based on whole-genome analysis to evaluate the chromosome instability (CIN) value and identified the potential relationship between CIN and prognosis of breast cancer patients with germline-*BRCA1* mutation.

**Materials and methods:**

Sanger sequencing or a 98-gene panel sequencing assay was used to screen for *BRCA1* germline small mutations in 1151 breast cancer patients with high-risk factors. MLPA assay was employed to screen *BRCA1* large genomic rearrangements in familial breast cancer patients with *BRCA1* negative for small mutations. Thirty-two samples with unique *BRCA1* germline mutation patterns were further subjected to CIN evaluation by LPWGS (low-pass whole-genome sequencing) technology.

**Results:**

Firstly, 113 patients with germline *BRCA1* mutations were screened from the cohort. Further CIN analysis by the LPWGS assay indicated that CIN was independent from the mutation location or type of *BRCA1*. Patients with high CIN status had shorter disease-free survival rates (DFS) (HR = 6.54, 95% CI 1.30–32.98, *P* = 0.034). The *TP53* copy loss was also characterized by LPWGS assay. The rates of *TP53* copy loss in CIN high and CIN low groups were 85.71% (12/14) and 16.67% (3/18), respectively.

**Conclusion:**

CIN-high is a prognostic factor correlated with shorter DFS and was independent with the germline *BRCA1* mutation pattern. Higher CIN values were significantly correlated with *TP53* copy loss in breast cancer patients with germline *BRCA1* mutation. Our results revealed a reliable molecular parameter for distinguishing patients with poor prognosis from the *BRCA1*-mutated breast cancer patients.

## Introduction

*BRCA1* is the most critical breast cancer hereditary susceptibility gene, which encodes homonymic proteins that serves in DNA repair processes during cellular stress. *BRCA1* mutation could lead to inactivation of the homologues recombinant repair (HRR) mechanism and induce chromosome instability (CIN). Although been widely researched, the clinical interests of germline *BRCA1* mutation in breast cancer prognosis have not been clearly described, with numerous studies reporting inconsistent results [1–5].

CIN serves as an important driver for carcinogenesis since it can lead to dramatic chromosomal structure malformation in cancer [6, 7]. CIN can be subdivided into two major categories: (1) gain and/or loss of the whole copy of chromosomes, which are also termed as numerical-CIN or “aneuploid”; (2) regional alterations on some chromosomes, which are termed as “structural-CIN” that include amplifications, deletions, inversions, and translocations of DNA fragments [8]. In breast cancer, numerous researchers have proved that CIN can predict the biological behavior and clinical outcomes [9–11]. These remind us the potential value of CIN in determine the prognosis of patients with germline *BRCA1* mutation, which has not been detailly investigated yet.

Recently, the limitation of breast cancer CIN evaluation remains to be the methodology since standardized protocol is still unavailable. Although lots of technologies have been introduced, the most popular approach to evaluate CIN remains nucleotide in situ hybridization, which is only a restricted reflection of the global genomic disorder [12–15]. Over recent years, the development and popularization of the Next Generation Sequencing (NGS) provided a novel solution for global CIN assessment: low-pass whole-genome sequencing (LPWGS), which depict the global genomic alteration rather than a single chromosome segment valuated by nucleotide hybridization.

Our current investigation explored the potential prognostic value of CIN in breast cancer patients with germline *BRCA1* mutation as well as the possible molecular mechanism. We also established a scoring system to quantify the CIN status based on the LPWGS method.

## Materials and methods

### Study population and samples

We reviewed the clinical data of patients who were admitted to Cancer Hospital of the University of Chinese Academy of Sciences (Zhejiang Cancer Hospital) from 2008 to date. Patients with pathological diagnosis of breast cancer and any one of the following risk factors were enrolled: (1) early onset of breast cancer with age ≤ 40 years; (2) TNBC with age ≤ 50 years; (3) with bilateral breast lesions or ≥ 2 ipsilateral breast lesions; (4) male breast cancer; (5) familial history of either ≥ 1 close relative with breast cancer (age of onset ≤ 50 years), ≥ 1 close relative with ovarian cancer (any age of onset) or ≥ 2 close relatives with breast cancer, prostatic cancer (Gleason score ≥ 7 or with terminal metastasis) or pancreas cancer at any age of onset. Peripheral blood samples were collected from all participants in EDTA tubes and stored at -80 °C. Their tissue blocks were collected for further DNA extraction after reviewing the HE slides.

The study was approved by Ethics Committee of Cancer Hospital of the University of Chinese Academy of Sciences (Zhejiang Cancer Hospital). Written informed consent was obtained from all recruited subjects.

### Clinical and pathological data collection

The clinicopathological information of all enrolled breast cancer patients, including age at diagnosis, histologic type and grade, molecular type, and status of ER, PR, and HER2 were collected. ER, PR, and HER2 expression were determined by immunohistochemistry (IHC). Cases with HER2 “2 + ” scores were further confirmed by fluorescence in situ hybridization (FISH). The follow-up information was collected either from medical records of regular follow-up examination or via telephone. The follow-up information included the time of follow-up, post-surgery treatment, time of recurrence and metastasis, metastatic status, and date and cause of death. Disease-free survival (DFS) was analyzed.

### BRCA1 germline mutations analysis

The DNA samples were prepared from the peripheral blood samples. Three phases of mutation analysis were performed. First, Sanger sequencing was performed on 133 unrelated familial breast and/or ovarian cancer (FBOC) cases using a total of 32 pairs of primers covering entire coding regions and exon–intron boundaries of *BRCA1*. Second, a 98-gene panel sequencing assay was employed to screen for mutations of breast cancer susceptibility genes in 1018 cases, which included *BRCA1*. The NGS panel was adopted for the NEBNext Direct sequencing technology (New England Biolabs, Ipswich, MA). Third, if FBOC cases were identified as negative for *BRCA1* small mutations by sequencing assays, large genomic rearrangements (LGRs) of *BRCA1* were screened by Multiplex ligation-dependent probe assay (MLPA) assay using the SALSA P002 kit (MRC-Holland, Amsterdam, the Netherlands). The variant classification was performed according to the American College of Medical Genetics (ACMG) guidelines.

### Tumor samples analysis by LPWGS assay

DNA was extracted from breast cancer FFPE tissues with *BRCA1* germline pathogenic or likely pathogenic mutation and analyzed by Illumina X10. At least 10 M paired reads were collected for each sample. The reads were mapped to human reference genome hg19. Genomic coverage was then counted using software samtools mpileup. Next, the average coverage was calculated for each 200 k bin. Z-scores for each bin were then normalized with Z-score using the following formula:

1 $${\mathrm{coverage}}_{\mathrm{normalized}}=\frac{{\mathrm{coverege}}_{\mathrm{raw}}-\mathrm{mean}({\mathrm{coverage}}_{\mathrm{controls},\mathrm{ raw}})}{\mathrm{stdev}\left({\mathrm{coverage}}_{\mathrm{controls},\mathrm{ raw}}\right)}.$$

Circular binary segmentation (CBS) algorithm from R package DNACopy was then used to identify significant genomic breakpoints, and copy number changed genomics segments.

R package ‘DNACopy’ was used to analyze copy number changes. A *P* value of < 0.05 was considered as statistically significant binary segmentation. The absolute segment value was used for further analysis. The sensitivity and specificity of UCAD were estimated by Receiver Operating Characteristic (ROC) curves. For categorical variables, the chi-square test was used as appropriate.

Proportion trend tests were used to analyze the associations between clinicopathological UCAD screening positivity and clinicopathological parameters. Data are reported as means and standard deviations, medians and interquartile ranges, and hazard ratios or odds ratios with 95% confidence intervals, as appropriate. Missing data were removed from the analyses. All analyses were performed with the use of R software, version 3.4.3 (R Foundation for Statistical Computing).

### CIN evaluation and TP53 copy loss identification

To evaluate the CIN status, *Z* scores of coverage for each chromosomal arm were estimated using the following formula,

2 $${Z}_{\mathrm{chr}}=\frac{{\mathrm{coverege}}_{\mathrm{chr},\mathrm{tumor}}-\mathrm{mean}({\mathrm{coverage}}_{\mathrm{chr},\mathrm{controls}})}{\mathrm{stdev}\left({\mathrm{coverage}}_{\mathrm{chr},\mathrm{controls}}\right)}.$$

CIN scores were summarized by formula-1, CIN = sum (*L*_chr_ * *Z*_chr_), where *L*_chr_ indicated the length of the chromosome segments and *Z*_chr_ presented the *Z* score of the segment. The cut-off *Z* score that separating the CIN low and high groups were determined as 4000 in our research.

To evaluate whether there was copy loss of *TP53* gene, *Z* scores of coverage for each chromosomal arm were estimated by the following formula,

3 $${Z}_{\mathrm{TP}53}=\frac{{\mathrm{coverege}}_{TP53,\mathrm{tumor}}-\mathrm{mean}\left({\mathrm{coverage}}_{\mathrm{TP}53,\mathrm{controls}}\right)}{\mathrm{stdev}\left({\mathrm{coverage}}_{\mathrm{TP}53,\mathrm{controls}}\right)}$$

Any sample with Z_TP53_ <  = − 3 indicates *TP53* gene copy loss.

### Statistical analysis

Single parameter and multiple survival analyses were performed with Cox regression and DFS rates were calculated with the Kaplan–Meier method. The correlation analyses between all of the parameters were performed by Fisher exact test. All statistical analyses were performed using R software (Ver 4.0.2).

## Results

### BRCA1 mutation frequency in breast cancer patients with high-risk factors

A total of 1151 breast cancer patients having one or more high-risk clinical factors were enrolled for BRCA1 germline mutation screening. 113 cases with the *BRCA1* mutation were identified, including five *BRCA1* LGRs and 108 small pathogenic or likely pathogenic mutations. The overall mutation rate of *BRCA1* was 9.8% (113/1151) in our cohort. The highest subgroup included patients with FBRC, which accounted for 16.5% (84/510). Detailed *BRCA1* mutation frequencies in all these sub-groups are listed in Table 1.

**Table 1:** *BRCA1* mutation rate in patients with predisposing factors

Clinicopathological characteristics	Case number	Case number with *BRCA1* mutation	*BRCA1* mutation rate (%)
Familial Breast Cancer(FBRC)	510	84	16.5
Triple Negative Breast cancer(TNBC)	162	18	9.9
Early Breast Cancer (EBC)	392	8	2.0
Bilateral Primary Breast Cancer(BPBC)	82	5	6.1
Male Breast Cancer (MBC)	5	0	0
Total	1151	113	9.8

### TP53 copy loss correlated with high CIN values in BRCA1 germline mutation patients

**Fig. 1:**
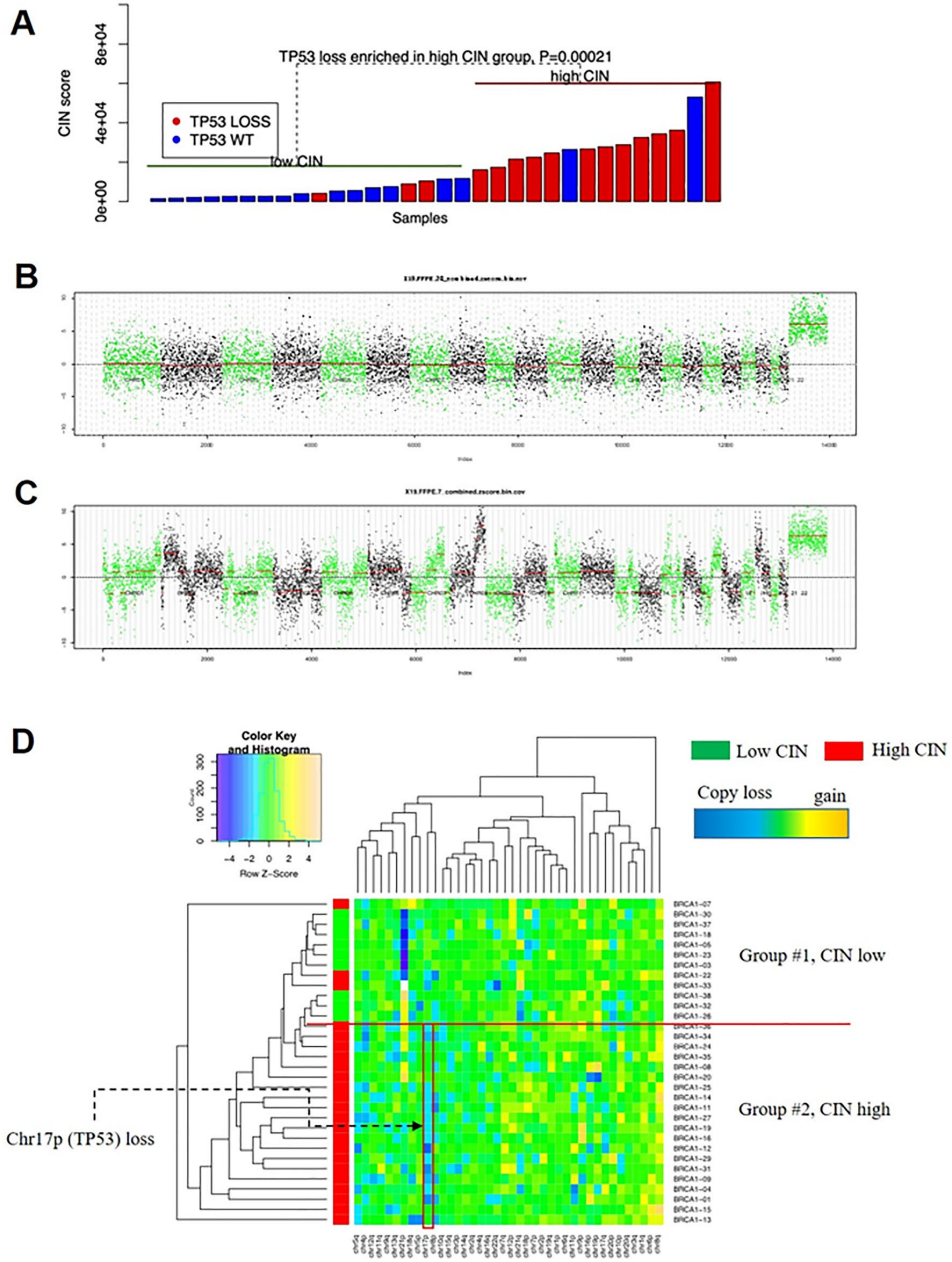
*TP53* copy loss significantly correlated with CIN value in *BRCA1*-mutated breast cancer. Distribution of cases with *TP53* loss in CIN low/high groups (**A**). The schematic illustration of CIN Z-score (**B**, **C**). The schematic illustration of the clustering (**D**)

Among the 113 *BRCA1*-mutated samples, 32 ones with unique *BRCA1* mutation characteristics were further analyzed for CIN and *TP53* status. The individual clinicopathological information as well as the molecular analysis results of these 32 cases are listed in Table 2. The LPWGS assorted 14 samples into CIN high group and the remaining 18 ones into CIN low group. The schematic illustration of CIN Z-scores and clustering are presented in Fig. 1B–D. The frequencies of *TP53* loss in the CIN high group and CIN low group were 85.71% (12/14) and 16.67% (3/18), respectively (Fig. 1A). These data indicated a significant correlation between CIN high phenotype and *TP53* loss (*P* = 0.00021). Further analysis of multiple single clinicopathological parameters indicated that *TP53* loss was a specific factor that determine the CIN status between the CIN low and high groups because other factors didn’t show significant impact (Table 3).

**Table 2:** Clinicopathological and molecular variation of the 32 patients

	Age of diagnosis	Pathological type^a^	Tumor size (cm)^b^	ER	PR	Her-2	Ki-67^c^	Vascular invasion	*BRCA1* mutation	BRCA1 amino acid change	*TP53* status	CIN Category
BRCA1-01	47	IDC	2	+	+	−	30%	Y	c.1209delT	p.Glu404AsnfsTer6	LOSS	High
BRCA1-03	40	IDC	1	−	−	−	NA	N	c.3295delC	p.Pro1099LeufsTer10	WT	Low
BRCA1-04	57	DCIS	1	+	−	+	30%	N	c.5251C > T	p.Arg1751Ter	LOSS	High
BRCA1-05	40	IDC	1	−	−	−	NA	N	c.5154G > A	p.Trp1718Ter	WT	Low
BRCA1-08	44	IDC	1	−	−	−	20%	N	c.223G > T	p.Glu75Ter	WT	Low
BRCA1-09	46	IDC	2	−	−	−	60%	N	c.4228delG	p.Glu1410LysfsTer5	LOSS	High
BRCA1-13	45	IDC	2	−	−	−	40%	N	c.5362delT	p.Gly1788ValfsTer5	LOSS	High
BRCA1-15	49	IDC	1	−	−	−	60%	N	c.5521delT	p.Ser1841ValfsTer2	WT	High
BRCA1-20	46	DCIS	1	+	−	−	10%	N	c.5137G > A	p.Asp1713Asn	WT	Low
BRCA1-32	50	IDC	2	−	−	−	45%	N	c.2971_2975delAAAAC	p.Lys991Ter	WT	Low
BRCA1-23	49	IDC	1	−	−	−	80%	N	c.4065_4068delTCAA	p.Asn1355LysfsTer10	WT	Low
BRCA1-37	37	IDC	2	−	−	−	20%	N	c.302-2A > C	–	WT	Low
BRCA1-38	33	IDC	2	+	−	−	70%	N	Exon13-14dup	–	WT	Low
BRCA1-26	54	IDC	2	−	−	−	NA	N	c.140G > T	p.Cys47Phe	WT	Low
BRCA1-30	29	IDC	1	−	−	−	70%	N	c.5470_5477delATTGGGCA	p.Ile1824AspfsTer3	WT	Low
BRCA1-18	44	DCIS	2	−	−	−	10%	N	c.981_982delAT	p.Cys328Ter	WT	Low
BRCA1-34	32	IDC	2	−	−	−	NA	Y	c.5161C > T	p.Gln1721Ter	LOSS	Low
BRCA1-33	40	IDC	2	+	+	−	15%	Y	c.4063_4066delAATC	p.Asn1355LysfsTer10	LOSS	Low
BRCA1-36	38	IDC	2	−	−	−	90%	Y	c.5468-1_5474delGCAATTGG	–	WT	Low
BRCA1-22	50	IDC	1	+	+	−	40%	N	c.4228delC	p.Glu1410LysfsTer5	WT	Low
BRCA1-24	55	IDC	2	−	−	−	–	N	c.2110_2111delAA	p.Asn704CysfsTer7	WT	Low
BRCA1-16	52	IDC	2	−	−	−	85%	N	c.5470_5477delATTGGGCA	p.Ile1824AspfsTer3	LOSS	High
Sample ID	Age of diagnosis	Pathological type	Tumor size (cm)	ER	PR	Her−2	Ki-67	Vascular invasion	*BRCA1* Mutation	BRCA1 amino acid change	*TP53* status	CIN Category
BRCA1-35	36	IDC	2	−	+	−	90%	N	c.4453_4454delAC	p.Thr1485GlnfsTer2	WT	Low
BRCA1-31	39	IDC	NA	NA	NA	NA	NA	N	c.3780delAG	p.Leu1260PhefsTer6	LOSS	Low
BRCA1-19	42	IDC	1	−	−	−	35%	N	Exon5-7dup	/	LOSS	High
BRCA1-27	58	IDC	1	−	−	−	NA	N	c.1465G > T	p.Glu489Ter	LOSS	High
BRCA1-29	47	IDC	3	+	−	−	0.6	Y	c.3770_3771delAG	p.Glu1257GlyfsTer9	LOSS	High
BRCA1-12	35	IDC	3	−	−	−	80%	N	c.4864A > T	p.Lys1622Ter	LOSS	High
BRCA1-07	48	IDC	1	−	−	−	70%	N	c.1058G > A	p.Trp353Ter	WT	High
BRCA1-11	39	IDC	1	−	−	−	70%	Y	c.4564delT	p.Tyr1522ThrfsTer26	LOSS	High
BRCA1-25	40	IDC	1	−	−	−	80%	Y	c.5137G > A	p.Asp1713Asn	LOSS	High
BRCA1-14	20	IDC	1	−	−	−	80%	N	c.537C > A	p.Tyr179Ter	LOSS	High

^a^DCIS, Ductal Carcinoma in Situ; IDC, Invasive Ductal Carcinoma

^b^ Tumor size (T): “1” indicates T ≤ 2 cm, “2” indicates T ≥ 2 cm and < 5 cm, “3” indicates T ≥ 5 cm. (c) *NA* not available

**Table 3:** Single parameter analysis of multiple clinicopathological factors between CIN High and CIN Low groups

	CIN High	CIN Low	*P* value
	(*N* = 14)	(*N* = 18)	
BRCA1 mutation			
Frameshift	7	9	0.82
Stopgain	5	4	
Splicing	0	2	
Missense	1	2	
LGR	1	1	
*TP53*			
Wild type	2	15	**0.00021**
Alteration	12	3	
Personal cancer history			
Only one breast cancer	10	13	1
≥ 2 primary breast cancers	4	5	
Concurrent ovary cancer	6	4	
Family history			
Breast cancer family history	5	12	0.73
Ovarian cancer family history	2	4	
Pancreatic cancer family history	0	2	
No cancer family history	6	9	
Onset of breast cancer (year)			
≤ 40	4	9	0.29
> 40	10	9	
Tumor size (cm)			
DICS	1	1	0.26
≤ 2	6	6	
> 2 and ≤ 5	4	10	
≥ 5	2	0	
Unknown	1	1	
Pathological type			
DICS	1	2	1
Invasive ductal carcinoma	13	15	
Other	0	1	
Pathological grade			
I	0	1	0.79
II	3	2	
III	5	7	
Unknown	6	8	
Vascular invasion			
Positive	4	3	0.67
Negative	10	15	
ER and/or PR			
Positive	1	4	0.37
Negative	11	13	
Unknown	2	1	
HER2			
Negative	14	17	1
Unknown	0	1	
Lymph node			
0	9	9	0.39
1–3	2	7	
4–9	2	2	

*LGR* large genomic rearrangement, *DCIS* ductal carcinoma in situ, *ER* estrogen receptor, *PR* progesterone receptor, *HER2* human epidermal growth factor receptor 2. Note: Among these parameters, only the *TP53* alteration presented significant difference between CIN High and CIN Low groups.

### High CIN leads to poor survival in breast cancer patients with BRCA1 germline mutations

**Fig. 2:**
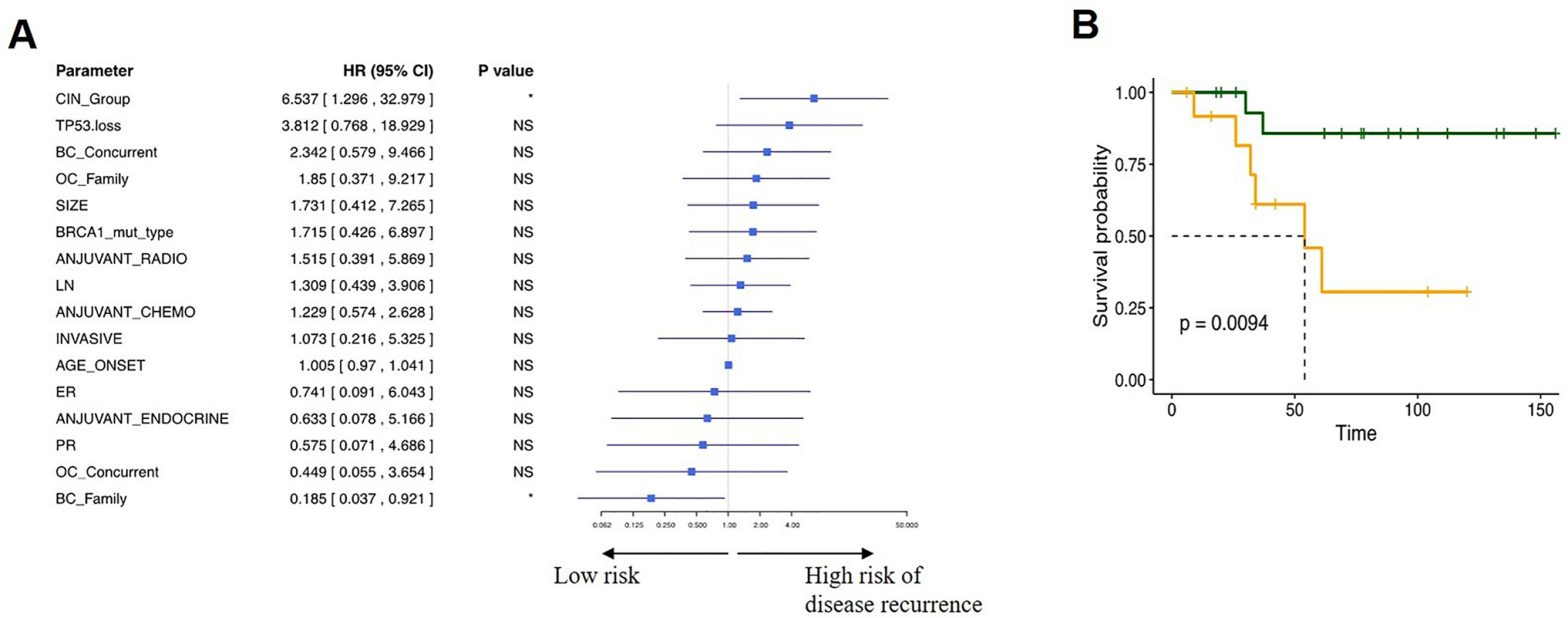
CIN status can predict the clinical outcomes of *BRCA1*-mutated breast cancer. The hazard ratio and the survival probability are analyzed by single parameter forest plots (**A**) and Kaplan–Meier analysis (**B**). High CIN predicts shorter disease-free survival (HR = 6.54, 95% CI 1.30–32.98, *P* = 0.034)

To identify the factor which impact prognosis of breast cancer with *BRCA1* germline mutation, we sought to determine the value of CIN in survival. Both single (Fig. 2A and Table 3) and multiple parameter analyses (Table 4) with Cox regression indicated that high CIN value led to poor survival (*P* = 0.013, HR = 6.537, and *P* = 0.044, HR = 5.99, respectively,). Kaplan–Meier analysis further revealed a decreased DFS in high-CIN patients (*P* = 0.0094, Fig. 2B).

**Table 4:** Multiple parameter survival analyses

Risk Factors	*P* value	HR	95% Lower	95% Upper
CIN high	**0.044**	5.99	2.46	14.6
Family breast cancer history	**0.035**	0.130	0.0493	0.342

Note: CIN high predicts worse survival independent of family breast cancer history in *BRCA1*-mutated breast cancer

### BRCA1 mutation profile did not impact the CIN value

**Fig. 3:**
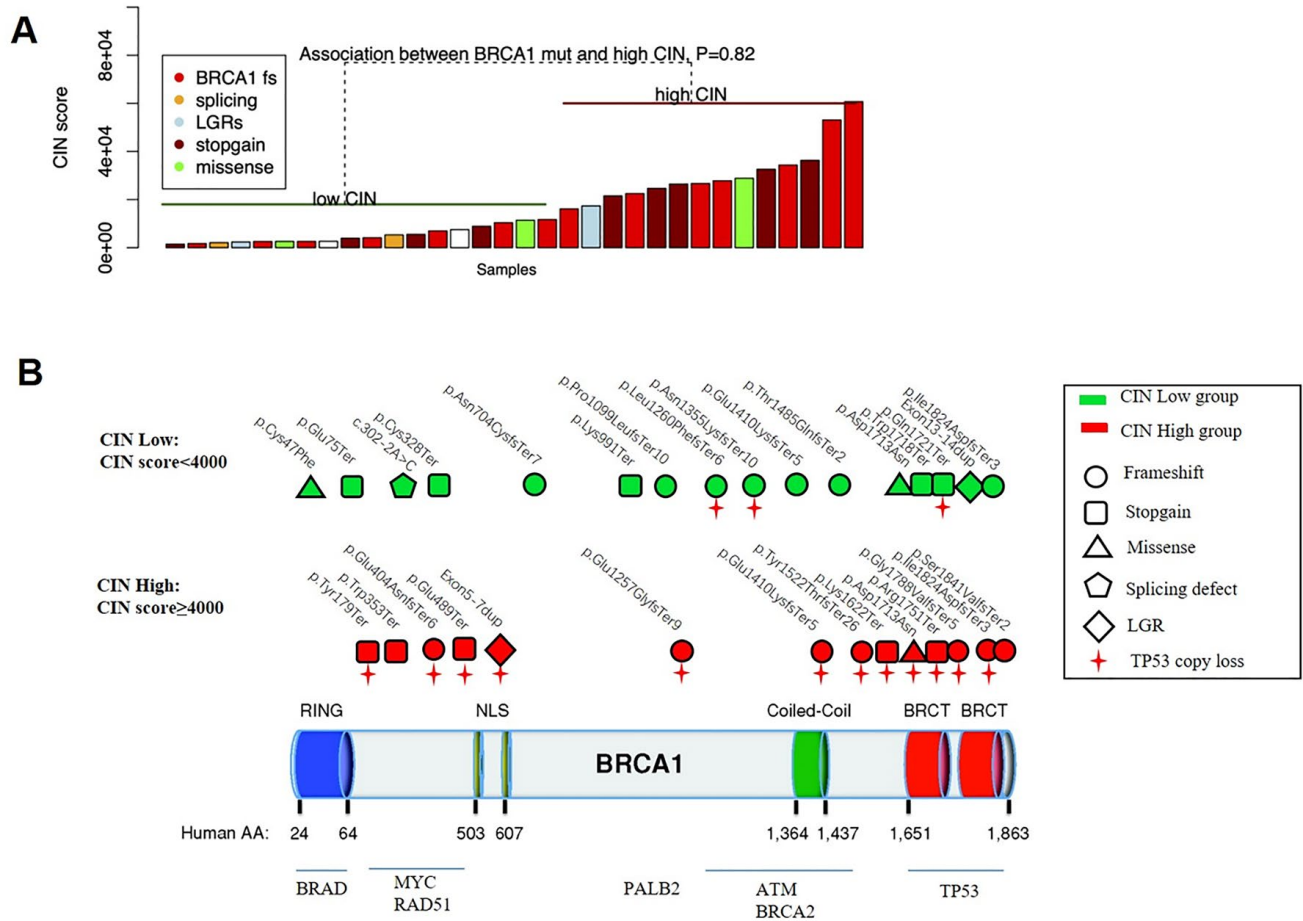
*BRCA1* mutation type and location did not infect the CIN of breast cancer. *BRCA1* mutation types within the CIN high and low groups, no significant difference was observed between these two groups (*P* > 0.05) (**A**). BRCA1 mutations of the two groups mapped to functional domains of *BRCA1* (**B**)

The relationships between CIN status and *BRCA1* mutation types (frame shift, stopgain, splicing defects, missense and large genomic rearrangement), mutation positions (domain) were analyzed. Within all the 32 cases that were formerly identified to have unique *BRCA1* mutation profiles, no significant correlation was found between them (Fig. 3A). The schematic illustration of mutations distributed along the *BRCA1* gene, as well as CIN status and TP53 copy loss within the 32 cases was shown in Fig. 3B. These results suggested that *BRCA1* mutation profile may not impact the CIN status, whereas loss of TP53 function was associated with chromosome instability.

## Discussion

Over recent years, the popularization of the NGS technology provided another solution for CIN evaluation; nonetheless, the unification of CIN evaluation based on the NGS technology is necessary. Some researchers used gene panel sequencing assay to evaluate CIN. In 2019, Lee et al.evaluated CIN scores based on a gene panel including 170 common cancer drivers performed by the NGS and identified the relationship between this“panel-based-CIN” and CEP 17 copy gain [14]. Although this method is better than FISH, it can still only provide a sidelight instead of global CIN status. Herein, we report a novel method to preform CIN evaluation by LPWGS due to its advantage in whole genomic coverage and sensitivity in detecting large size DNA alterations. This method has been utilized by some previous studies to clinically identify the cytogenetics [16, 17]. In the meantime, circulating Cell-free DNA evaluation by this method has also been reported and thought to be valuable in predicting the prognosis or chemotherapy resistance in non-small cell lung cancer and breast cancer [18–20]. Based on these reports, LPWGS is thought to be a reliable method in determine large segments alterations of cancer genomes. Here in our present research, we tried this method to analyze breast cancer CIN status to overcome the limitations of the former CIN evaluation method based on FISH.

Since we included high-risk clinical factors, the mutation frequency of *BRCA1* in our cohort was 9.8% (113/1151), which was more than in unselected breast cancer populations, accounting for approximately 5%, according to the Cancer Genome Atlas [4, 5, 10]. However, the prognosis of this *BRCA1*-mutation-subgroup remains to be elusive since various clinical trials and researches reported inconsistent results [21–24]. According to two large-scale clinical researches based on Chinese breast cancer patients published in 2017, the authors reported negative results because *BRCA1* mutation was not significantly correlated with poor prognosis in multivariate analysis [4, 5]. Thus, the identification of reliable molecular factors for sub-classification of patients with a *BRCA1* mutation is of great value to develop potential efficient molecular therapy for this specific population. It has been reported that the CIN level is correlated with the prognosis in breast cancer [14, 25]. In our research, we demonstrated that *BRCA1* germline mutation breast cancer patients with high CIN values suffered from shorter DFS (Fig. 2B). This suggests that CIN determined by the LPWGS method may be used as a valuable prognosis factor in *BRCA1*-mutated breast cancer patients.

Our data also revealed the potential mechanism leading to CIN elevation in breast cancer with *BRCA1* germline mutation. In our cohort, *TP53* copy loss (SNV of *TP53* was not included) was related to the high CIN phenotype. Among the 32 cases, 15 ones were with *TP53* copy loss (12 of 14 cases in CIN high group and 3 of 18 cases in CIN low group, *P* = 0.00021). This may be explained with the nature of CIN, which was caused by a failure in repairing DNA double strain break (DSB) due to the germline or somatic mutation of some genomic homeostatic genes, including homologous recombination repair (HRR) genes (e.g., *BRCA1, BRCA2, PALB2, RAD51C, BRIP1*) and genome caretaker genes such as *TP53*. Aberrant expression of *TP53* disables cellular response to DNA damage on multiple levels [26]. Cancer cells with *TP53* copy loss cannot arrest their cell cycle, which is necessary for proper DNA damage fixation, thus leading to accumulated large DNA fragments alterations such as translocations, duplications, and deletions [27, 28]. Consequently, *TP53* copy loss leads to dramatically increased CIN and cancer malignancy.

Previous studies has found that the *BRCA1* mutation related genomic instability was at least partially dependent on abnormal TP53 activation, demonstrating that TP53 participated in the biological processing of these signal transduction [29, 30]. In one study, Cao et al. found that stem cells with *BRCA1* deletion presented senescence characteristics and they may underwent malignant transformation with TP53 copy loss at the mean time [31]. Thus, *BRCA1*—*TP53* axis ensures the stability of somatic genomics, and prevent oncogenic progression. The potential mechanism of *TP53* loss in breast cancer with *BRCA1* germline mutation need to be investigated.

The mutation spectrum of the *BRCA1* is extremely complicated. Various mutation sites that involved almost all of its exons and introns have been identified, leading to all types of genetic alterations, including frameshift, missense, nonsense, inframe insertions and deletions, and splice altering mutations [32]. In the present study, we analyzed the *BRCA1* mutations in all our cases and obtained the mutation spectrum. Unfortunately, we did not find any convincing hot-spot exons for high CIN cases, not even in the three breast cancer cluster regions (BCCRs) previously identified [33]. These results indicated that CIN was independent of *BRCA1* mutation types and position. Despite the existence of numerous *BRCA1* variants, any pathogenic mutations on the *BRCA1* may be insufficient to accumulate chromosome instability.

## Conclusion

In summary, our research identified a novel whole-genome sequencing method to quantify the CIN and we found high CIN status was correlated with poor DFS as well as the copy loss of *TP53* in *BRCA1* germline mutation breast cancer, while the distribution of the mutation sites along the *BRCA1* gene did not affect the CIN value. According to these findings, application of the LPWGS methods will be valuable in CIN evaluation and can serve as a novel prognosis factor to predict the clinical outcome in patients with *BRCA1* mutation.

## Abbreviations

CIN: Chromosome instability
MLPA: Multiplex Ligation-dependent Probe Amplification
LPWGS: Low-pass whole-genome sequencing
*TP53*: Tumor protein p53
DFS: Disease-free survival
HER2: Human epidermal growth factor receptor 2
*BRCA1*: Breast cancer susceptibility gene 1
HRR: Homologues recombinant repair
TNBC: Triple-negative breast cancer
HE: Hematoxylin and eosin
FISH: Fluorescence in situ hybridization
IHC: Immunohistochemistry
FBOC: Familial breast and/or ovarian cancer
NGS: Next Generation Sequencing
ER: Estrogen receptor
PR: Progesterone receptor
FBRC: Familial breast cancer
EBC: Early breast cancer
BPBC: Bilateral Primary Breast Cancer
MBC: Male breast cancer
DSB: DNA double strain break
LGR: Large genomic rearrangement
DCIS: Ductal carcinoma in situ
